# *Helicobacter pylori* Affects the Antigen Presentation Activity of Macrophages Modulating the Expression of the Immune Receptor CD300E through miR-4270

**DOI:** 10.3389/fimmu.2017.01288

**Published:** 2017-10-12

**Authors:** Matteo Pagliari, Fabio Munari, Marta Toffoletto, Silvia Lonardi, Francesco Chemello, Gaia Codolo, Caterina Millino, Chiara Della Bella, Beniamina Pacchioni, William Vermi, Matteo Fassan, Marina de Bernard, Stefano Cagnin

**Affiliations:** ^1^Department of Biology, University of Padua, Padua, Italy; ^2^Department of Biomedical Sciences, University of Padua, Venetian Institute of Molecular Medicine (VIMM), Padua, Italy; ^3^Department of Molecular and Translational Medicine, Section of Pathology, University of Brescia, Brescia, Italy; ^4^CRIBI Biotechnology Center, University of Padua, Padua, Italy; ^5^Department of Medicine, University of Padua, Padua, Italy

**Keywords:** *Helicobacter pylori*, chronic inflammation, macrophages, microRNAs, CD300E, major histocompatibility complex class II

## Abstract

*Helicobacter pylori* (Hp) is a Gram-negative bacterium that infects the human gastric mucosa, leading to chronic inflammation. If not eradicated with antibiotic treatment, the bacterium persists in the human stomach for decades increasing the risk to develop chronic gastritis, gastroduodenal ulcer, and gastric adenocarcinoma. The lifelong persistence of Hp in the human stomach suggests that the host response fails to clear the infection. It has been recently shown that during Hp infection phagocytic cells promote high Hp loads rather than contributing to bacterial clearance. Within these cells Hp survives in “megasomes,” large structures arising from homotypic fusion of phagosomes, but the mechanism that Hp employs to avoid phagocytic killing is not completely understood. Here, we show that Hp infection induces the downregulation of specific microRNAs involved in the regulation of transcripts codifying for inflammatory proteins. miR-4270 targets the most upregulated gene: the immune receptor *CD300E*, whose expression is strictly dependent on Hp infection. CD300E engagement enhances the pro-inflammatory potential of macrophages, but in parallel it affects their ability to express and expose MHC class II molecules on the plasma membrane, without altering phagocytosis. This effect compromises the possibility for effector T cells to recognize and activate the killing potential of macrophages, which, in turn would become a survival niche for the bacterium. Taken together, our data add another piece to the complicate puzzle represented by the long-life coexistence between Hp and the human host and contribute with new insights toward understanding the regulation and function of the immune receptor CD300E.

## Introduction

*Helicobacter pylori* (Hp) is a Gram-negative bacterium recognized as human carcinogen in 1994 by the World Health Organization (1). Despite the incidence of the infection is globally declining, it remains high in several developing countries where it ranges between 50 and 70%, and the prevalence of infection is at least twofold higher in countries with high gastric cancer incidence (2). Many host, bacterial, and environmental factors act in combination to contribute to the precancerous cascade that, starting from the condition of chronic gastritis, leads to the development of gastric cancer. Hp is considered to be noninvasive because most of the bacteria reside in the mucous layer of the stomach in contact with the epithelium; however, it has been evidenced that the bacterium and its products can be in direct contact with immune cells in the lamina propria (3). As a consequence, infection with Hp results in a large influx of immune cells and in the induction of an adaptive immune response including both Th1 and Th17 components (4). Although it is clear that the ability of Hp to establish a persistent infection in its host relies on the delicate balance between inducing an adaptive immune response and escaping from it, one important aspect that remains to be fully elucidated is the role of the innate immune response in the inability of T lymphocytes to clear the bacterial infection.

It has been recently shown that during Hp infection phagocytic cells promote high Hp loads rather than contributing to bacterial clearance (5); in accordance, computational modeling of immune responses against Hp predicted that macrophages are central regulators of the mucosal immune response (6). How Hp manipulates the macrophage function remains to be established.

MicroRNAs (miRNAs) actively participate in the modulation of both innate and adaptive immune responses (7, 8). miRNAs are short non-coding RNAs (20–22 nt) that, upon the interaction with 3′-UTR of coding genes, inhibit their transduction or induce their degradation. The expression of several miRNAs, including miR-155 and -146a and the miR-200b, -200a, and -429 cluster, has been reported to be modulated by Hp in dendritic or gastric epithelial cells (9–12). Genome-wide analyses has been also performed in human gastric cancer cells (13) and in Hp-infected gastric mucosa (14), while a similar approach has not been applied yet to infected macrophages for which only miR-155 has been reported to be upregulated and acting as an inflammatory promoter (15).

In this work, we identified a complete list of miRNAs that are expressed in Hp-infected human macrophages. We evidenced that Hp infection reduces the expression of miR-4270 favoring the upregulation of the immune receptor CD300E. We also revealed that the engagement of CD300E compromises the expression of major histocompatibility complex class II (MHC-II) by macrophages resulting in the impairment of the antigen presentation ability.

Overall, our data provide evidence that mononuclear phagocytes are critical for the persistence of the bacterium in the stomach and corroborate the notion that these cells are central regulators of the mucosal immune response during Hp infection.

## Results

### Hp Infection of Macrophages Affects the Expression of miRNAs

**Figure 1:**
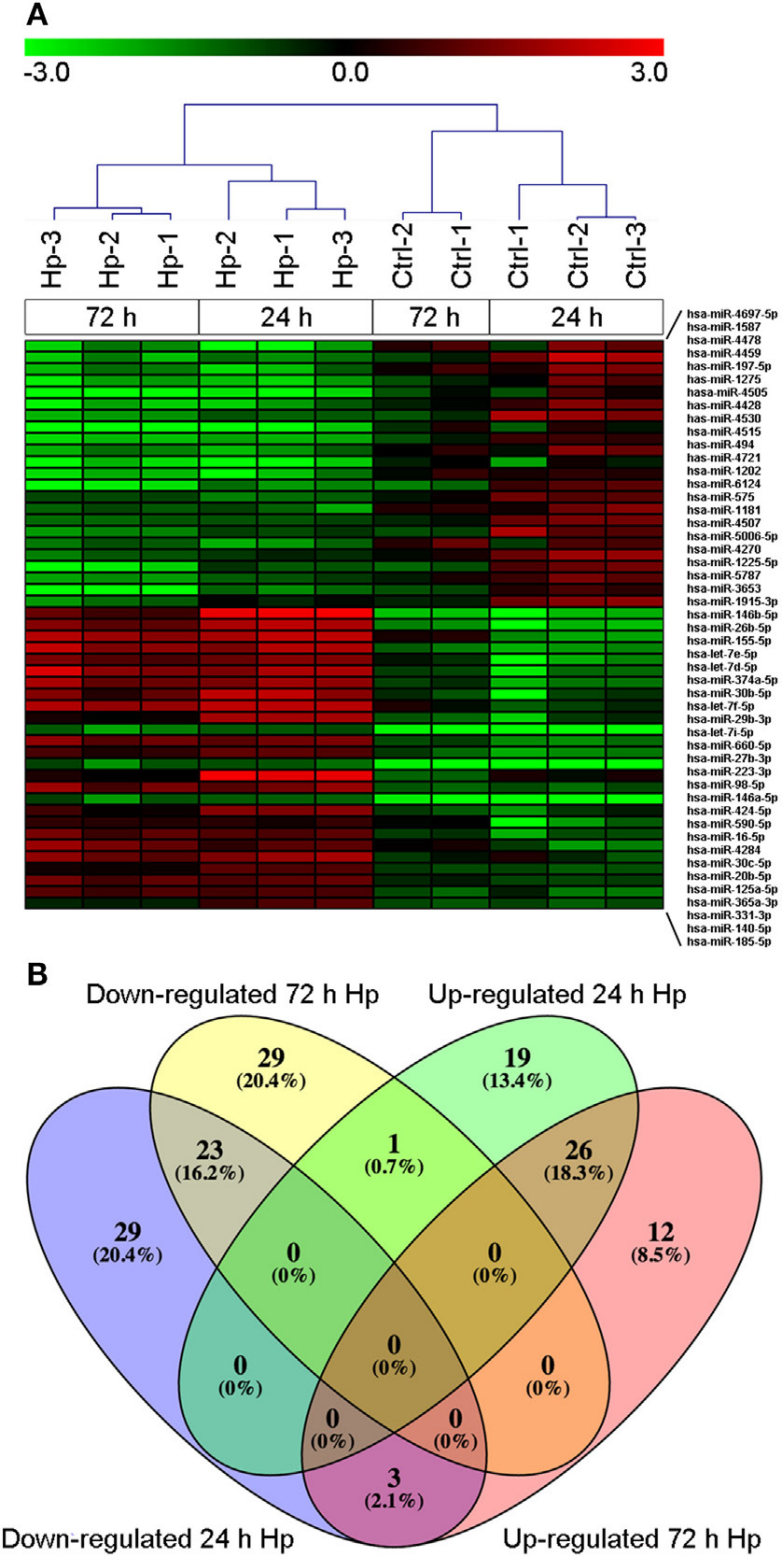
MicroRNA (miRNA) expression in macrophages infected with *Helicobacter pylori* (Hp). **(A)** Unsupervised cluster analysis of detected miRNAs separates uninfected from Hp-infected macrophages. Heat map represents the expression of differentially expressed miRNAs identified at 24 and 72 h of infection. Gene expression values are relative to the average expression of data collected at *T*_0_ and log scaled. **(B)** Venn diagram representing distribution of differentially expressed miRNAs.

With the aim of evaluating miRNA profile induced by Hp infection in macrophages, we adopted human monocyte-derived macrophages as cell model, and we exposed them to live bacteria. We found that, regardless of the infection, 270 of 2,600 detectable miRNAs were expressed in at least 50% of analyzed samples (Table S1 in Supplementary Material), and the most represented miRNA families were let-7, miR-17, miR-30, and miR-320 (Table S2 in Supplementary Material). Sample cluster analysis, based on the expression of detected miRNAs, revealed a differential expression of miRNAs in cells infected with Hp with respect to control cells (Figure 1A), suggesting that Hp infection actually modulates miRNA expression in macrophages. Fifty-five miRNAs were downregulated and 46 were upregulated after 24 h infection with Hp (Table S3 in Supplementary Material). Similarly, 53 miRNAs were downregulated and 41 were upregulated in macrophages exposed for 72 h to the bacterium (Table S4 in Supplementary Material). 26 miRNAs were upregulated and 23 were downregulated both at 24 and 72 h (Figure 1B; Tables S3 and S4 in Supplementary Material). Since Hp infection triggers a chronic inflammation, we reasoned that downregulated miRNAs, being permissive on the expression of their target genes, were the most interesting to investigate.

### Identification of miRNA Targets: miR-4270 Regulates the Expression of *CD300E*

**Figure 2:**
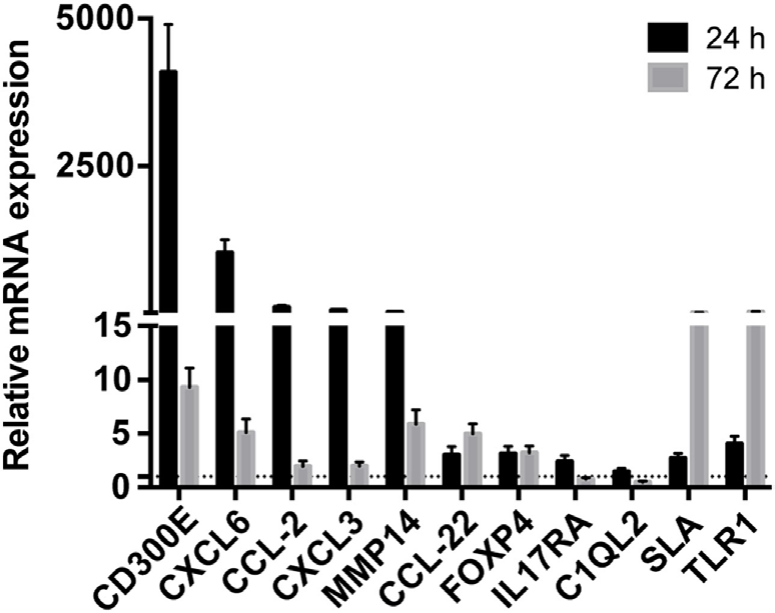
Validation of the expression of immunity-related genes in *Helicobacter pylori* (Hp)-infected macrophages. The expression of some upregulated genes in mouse macrophages infected with Hp was confirmed in human macrophages after the exposure to live bacteria for 24 and 72 h. All genes considered were upregulated either at 24 or 72 h of infection. The expression is relative to non-infected macrophages, and dashed line represents not differential expression.

Taking advantage from an already published work on mouse (16), we detected differentially expressed mRNAs in mouse macrophages infected with Hp and identified human orthologous presenting miRNA seed sequences in their 3′-UTR. 1,120 upregulated and 898 downregulated probes identified the coding genes activated and inhibited (843 and 763 genes, respectively) by the infection of mouse macrophages with Hp (Table S5 in Supplementary Material). 646 of 843 and 558 of 763 genes had human orthologous (Table S5 in Supplementary Material). Downregulated genes were mainly involved in the production of energy based on lipid oxidation, DNA replication, transcription, and repair (Table S6 in Supplementary Material), whereas a large proportion of upregulated genes were related to the immune response (Table S7 in Supplementary Material). The expression of several mRNAs, upregulated in mice macrophages infected with Hp, was validated in human macrophages infected with the bacterium (Figure 2). Among them, the most upregulated at 24 h postinfection were *CD300E, CXCL6, CCL2, CXCL3*, and matrix metallopeptidase 14 (*MMP14*), and some of which remained upregulated longer (72 h postinfection).

**Figure 3:**
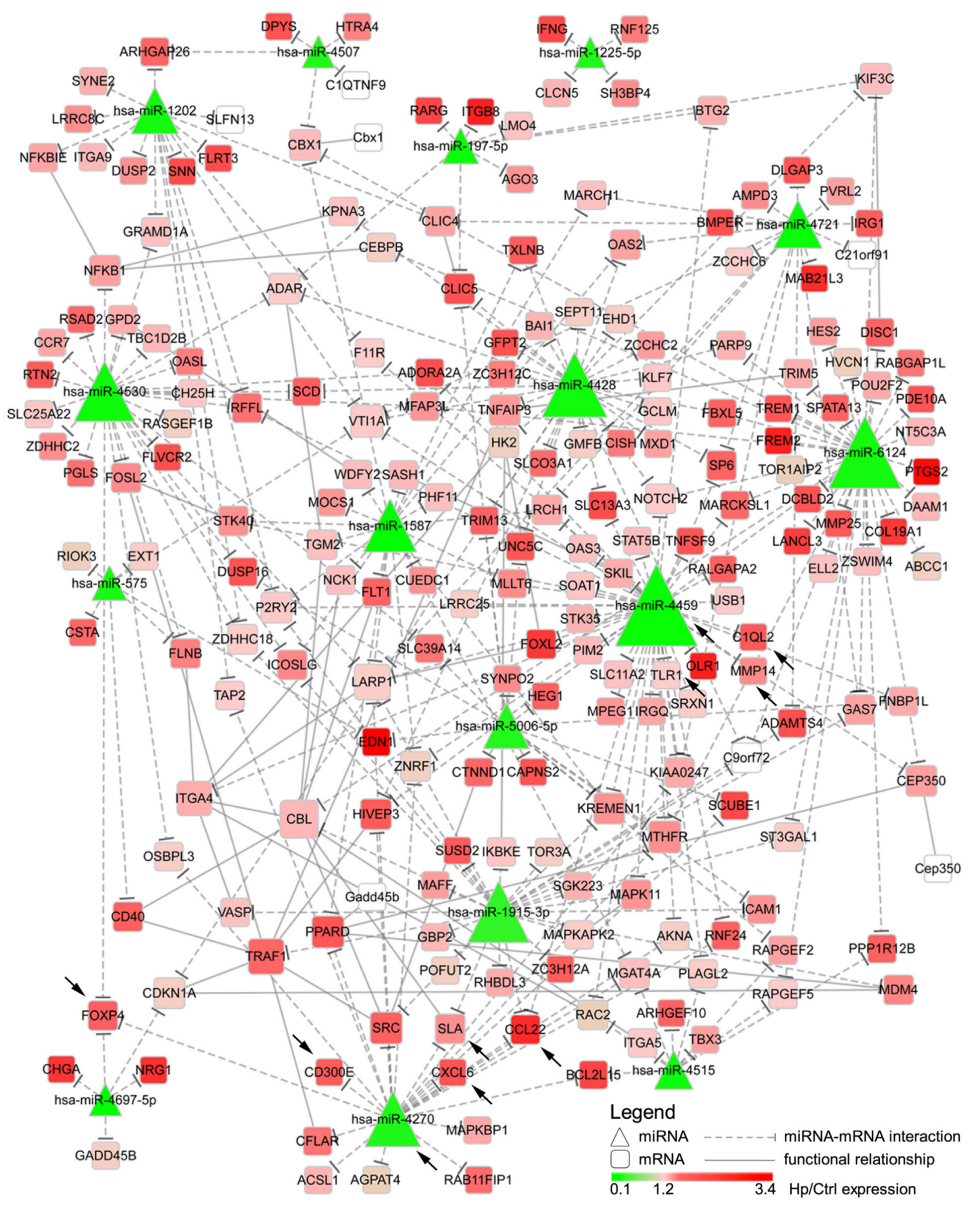
Integration of microRNAs (miRNAs) and mRNA targets. The network describes the interactions between miRNAs downregulated both after 24 and 72 h of *Helicobacter pylori* (Hp) infection and the predicted targets. The higher the node size is, the higher the number of edges connected with it is. Color scale mirrors the expression of miRNAs and genes in Hp-infected vs non-infected macrophages (Ctrl). Triangles represent miRNAs, whereas round squares refer to mRNAs. Dashes edges interconnect miRNAs with their targets, whereas filled edges describe functional relationships between different mRNAs. Arrows indicate genes, and miRNAs for whom the expression was confirmed in human derived macrophages by qRT-PCR.

**Figure 4:**
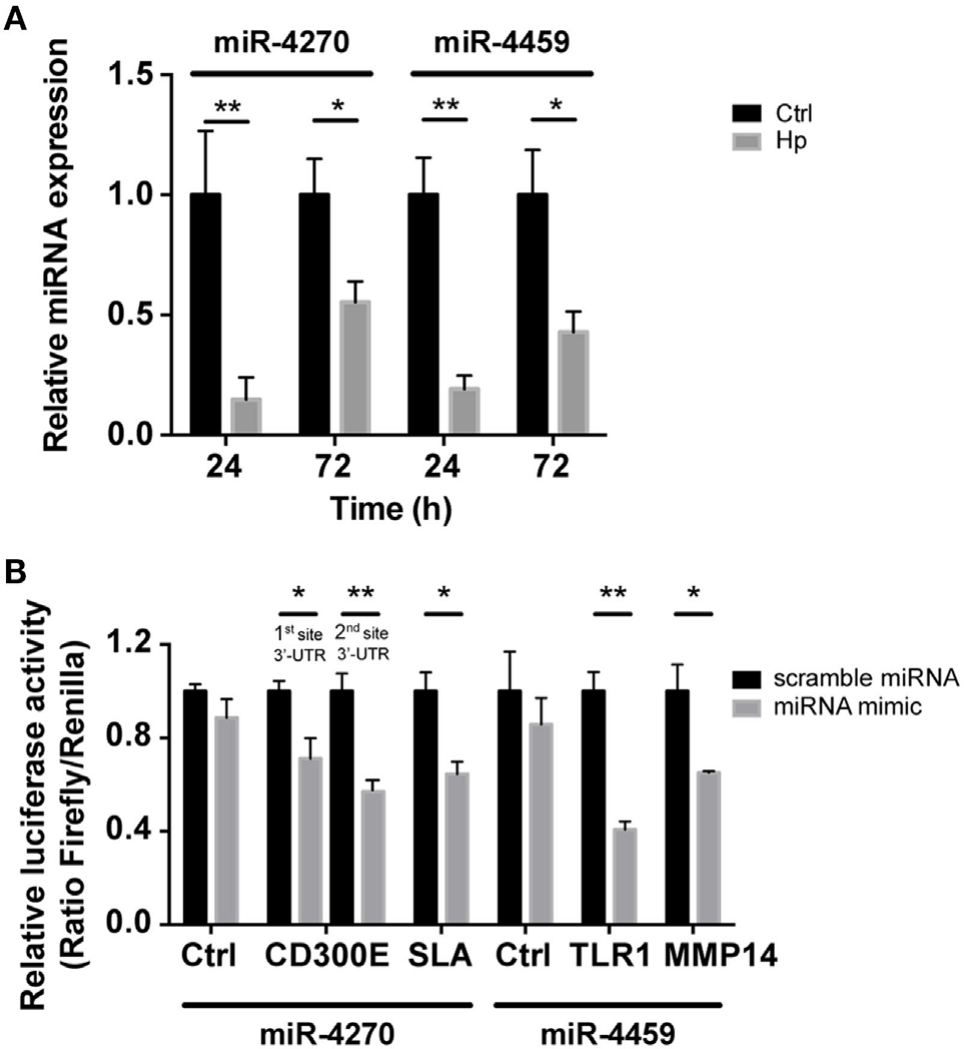
qRT-PCR analysis of miR-4270 and miR-4459 in *Helicobacter pylori* (Hp)-infected macrophages and validation of microRNA (miRNA)-target interaction. **(A)** miR-4270 and miR-4459 downregulation in Hp-infected human macrophages as assessed by qRT-PCR. miRNA expression was evaluated after 24 and 72 h of Hp infection. *p < 0.05; **p < 0.01. **(B)** Luciferase assay supports the interaction between miR-4459 and toll-like receptor 1 (*TLR1*) and matrix metallopeptidase 14 (*MMP14*) 3′-UTR, whereas miR-4270 was confirmed to interact with Src-like adaptor (SLA) and with two sites in the *CD300E* 3′-UTR. miRNA scrambled sequence and 3′-UTR control sequence (Ctrl) did not alter fluorescence for all the interactions tested.

We reasoned on the possibility that the upregulated genes were target of Hp-modulated miRNAs. Integrating the expression of miRNA predicted targets and downregulated miRNAs by Hp infection, we evidenced that 16 of the 23 miRNAs downregulated both at 24 and 72 h postinfection, actually found a correspondence in the upregulation of predicted targets (Figure 3). Six of 16 (miR-4530, -4428, -6124, -4459, -1915-3p, and -4270) occupied a central position in the regulation of inflammatory processes (Figure 3). We focused our attention on miR-4459 and miR-4270: the first because of the multiplicity of its targets and the second because predicted to target the most upregulated gene in macrophages infected with Hp, *CD300E*. For both miRNAs, we confirmed by qRT-PCR a downregulation of about 80% after 24 h and of about 50% after 72 h of infection (Figure 4A) and the activation of some of their targets (Figures 2 and 3), with respect to control cells. The interaction between miR-4459 and miR-4270 and two of their mRNA predicted targets were also confirmed by luciferase assay. In particular, miR-4459 was confirmed to directly interact with mRNA coding for toll-like receptor 1 and *MMP14*, whereas miR-4270 was confirmed interacting with mRNA coding for Src-like adaptor and *CD300E* (Figure 4B).

### Hp-Infected Macrophages Expose CD300E on the Plasma Membrane

**Figure 5:**
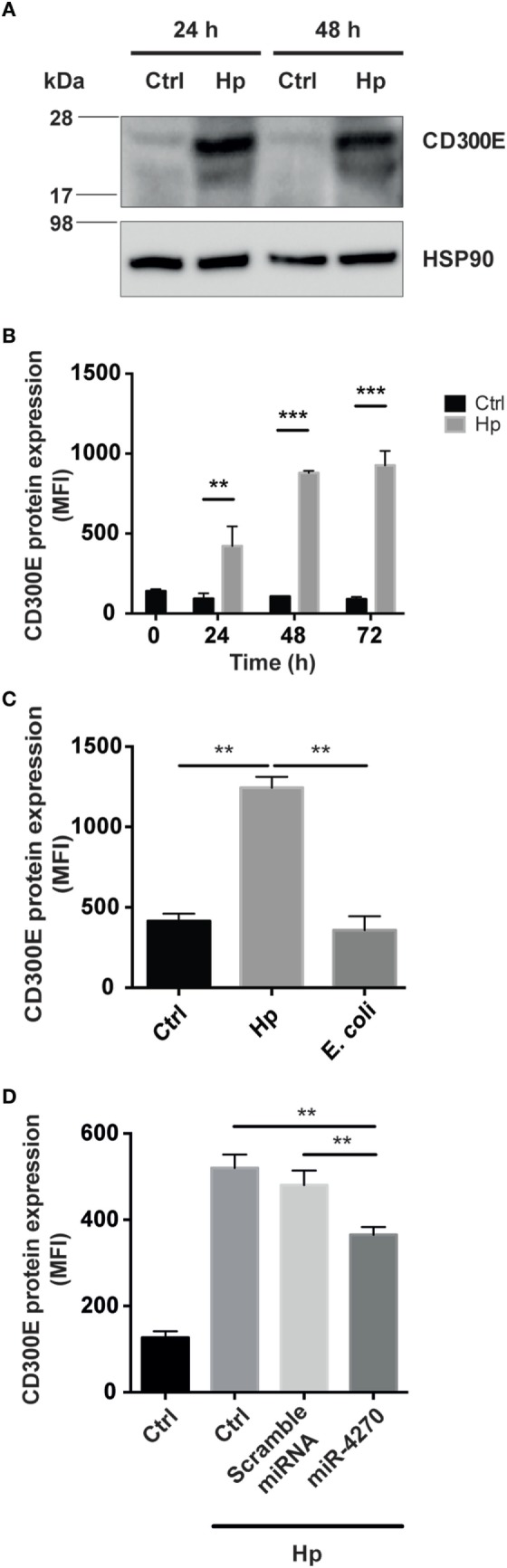
Macrophages infected by *Helicobacter pylori* (Hp) enhance the expression of CD300E. **(A)** Western blot of macrophages, before and after infection, developed with a polyclonal antibody anti-CD300E. Although a basal expression of CD300E is appreciable in not infected cells (Ctrl), it is highly induced upon Hp infection. The band between 17 and 28 kDa is consistent with the expected molecular weight of CD300E. **(B)** Time-dependent expression of CD300E in macrophages infected with Hp, evaluated by cytofluorimeter. **(C)** Expression of CD300E in macrophages infected with Hp or with *E. coli*. **(D)** Downregulation of CD300E expression in Hp-infected macrophages transfected with miR-4270 mimic. No effect was observed in macrophages upon transfection with a microRNA (miRNA) scramble. Transfection was performed 3 h after starting the infection, and cells were stained for CD300E after 24 h. Control (Ctrl) refers to cells transfected with control sequence. Data are shown as mean fluorescence intensity (MFI) ± SD of three independent experiments performed with three different cell preparations. Significance was determined by Student’s *t*-test. **p < 0.01; ***p < 0.001.

CD300E is a surface molecule, originally termed immune receptor expressed by myeloid cells-2. *In vitro* evidence supports the notion that CD300E functions as an activating receptor capable of regulating the inflammatory and immune responses (17). CD300E is expressed at low level in muscle, lung, kidney, and adipose tissue (18), whereas it is upregulated in different cancers, namely, colorectal, stomach, liver, ovarian, and pancreatic cancer (19). Bone marrow is the tissue with the highest expression of CD300E (18) in accordance to the fact that myeloid dendritic cells and monocytes are the most highly expressing cells (20). The differentiation of monocytes into macrophages is accompanied by its downregulation (20). Thinking about this evidence, we were impressed by the finding that the infection of macrophages by Hp seemed to revert the expression of CD300E. In accordance, by comparing the expression of CD300E between uninfected macrophages and Hp-challenged macrophages, we revealed that Hp infection strongly increased the CD300E basal expression (Figure 5A).

Next, we assessed whether the CD300E molecule, overexpressed in infected macrophages, was exposed on the cell surface, as expected. As shown in Figure 5B, the exposure of macrophages to Hp resulted in a time-dependent accumulation of CD300E on the plasma membrane that, after 72 h of infection, was about 10 times over the level of mock cells. Notably, this effect was not recapitulated by macrophages exposed to a non-pathogenic *E. coli* (Figure 5C). Consistent with the above findings, transfection of macrophages with a miR-4270 mimic, significantly impaired the expression and the exposition on the plasma membrane of the immune receptor induced by the bacterium (Figure 5D).

### Macrophages Expressing CD300E Infiltrate the Mucosa of Hp-Induced Chronic Gastritis

**Figure 6:**
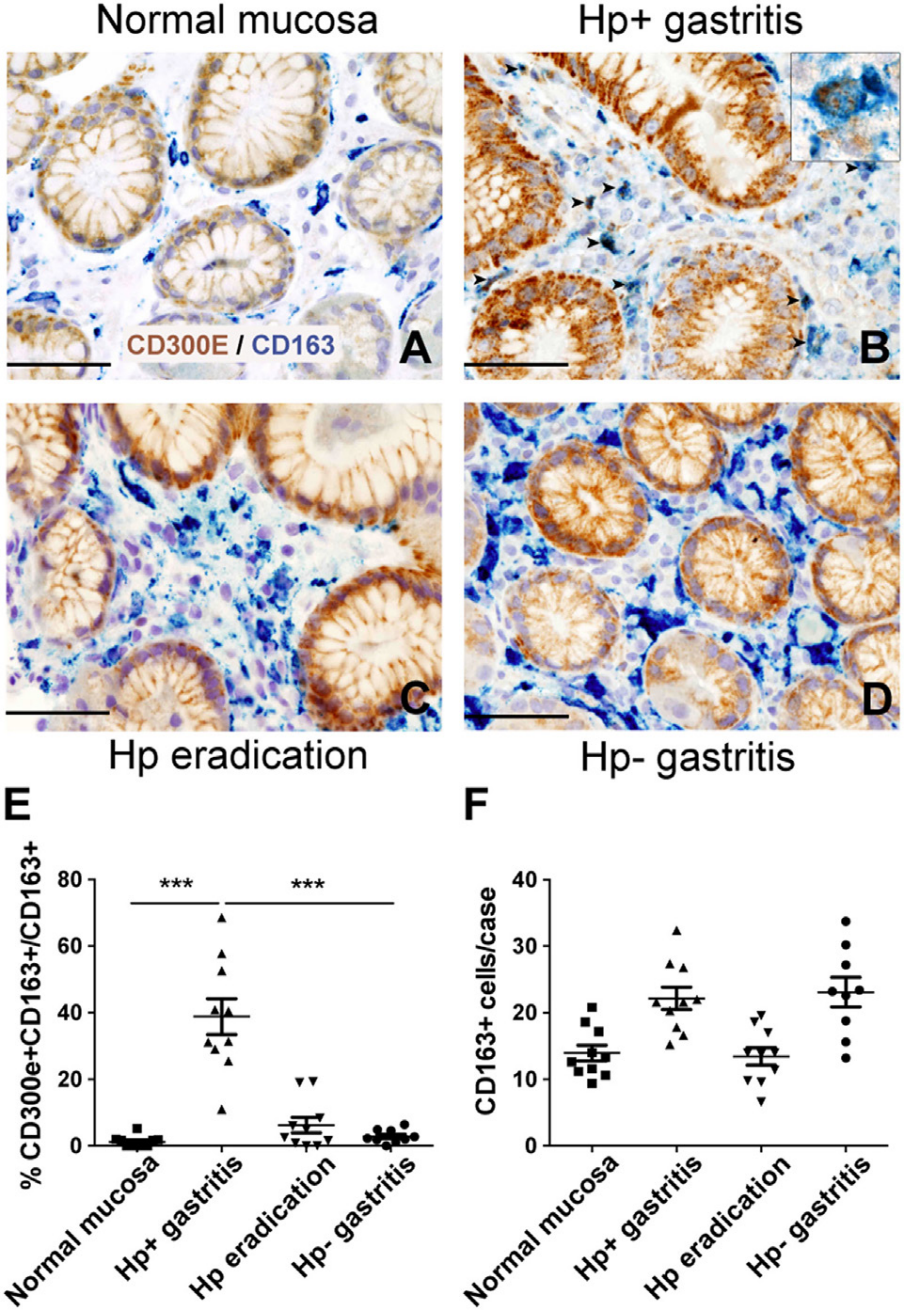
Expression of CD300E in macrophages infiltrating *Helicobacter pylori* (Hp) gastritis. Sections are from gastric biopsies **(A–D)** showing normal mucosa **(A)**, Hp^+^ gastritis, respectively, pre- **(B)** and post-Hp eradication **(C)**, and Hp^−^ gastritis **(D)**. Sections are stained for CD300E (brown) and CD163 (blue). The arrows and inset highlight CD163^+^CD300E^+^ double positive macrophages. Original magnification 400×, scale bar 50 mm. Inset 600×. **(E)** Graph showing the percentage of CD300E^+^ macrophages (CD163^+^) (***p = 0.0002 vs normal mucosa and ***p < 0.0001 vs Hp eradication and Hp^−^ gastritis). **(F)** Graph showing number of CD163^+^ macrophages in the cases analyzed.

The evidence that Hp promoted the expression of CD300E in macrophages prompted us to search for an *in vivo* correlation between Hp infection and the presence of macrophages expressing CD300E. We assessed the expression of the receptor on macrophages infiltrating the mucosa of human patients suffering from Hp gastritis. With respect to normal mucosa (Figure 6A), Hp-infected mucosa revealed that a significant proportion of macrophages, identified as CD163^+^ cells, were positive for CD300E (Figure 6B). The accumulation of CD300E^+^ macrophages strictly relies on the presence of the bacterium, since they were virtually absent in patients that underwent Hp eradication (Figure 6C) as well as in patients with Hp^−^ (negative) gastritis (Figure 6D). Notably, the fact that less CD163^+^/CD300E^+^ cells were present in the latter condition did not reflect a minor infiltration of macrophages with respect to the infectious gastritis, since they were numerically almost identical in the two conditions (Figures 6E,F).

### The Activation of CD300E Affects the Antigen Presentation Capacity of Macrophages

**Figure 7:**
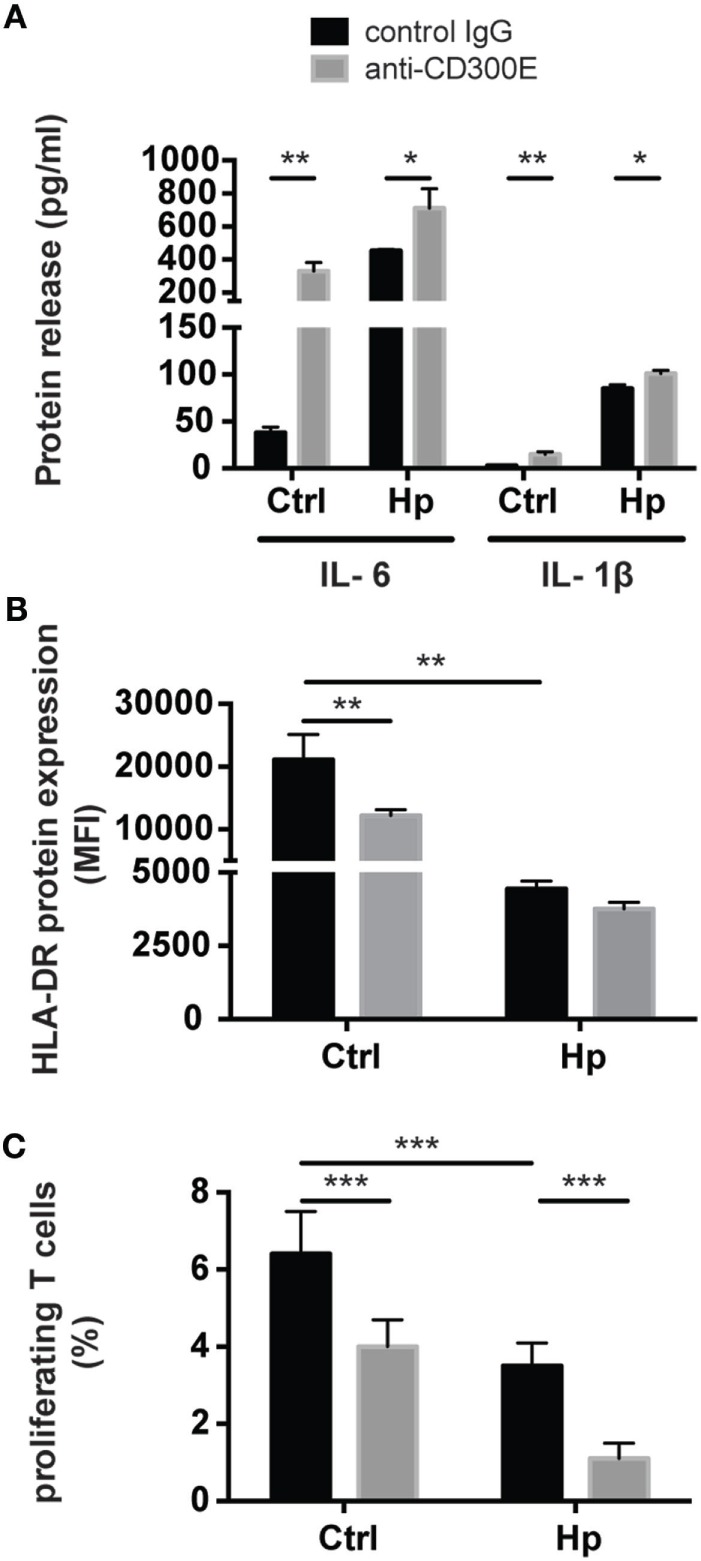
Effects of CD300E activation on macrophages. **(A)** Release of IL-1β and IL-6 by macrophages after the engagement of CD300E. **(B)** HLA-DR expression in macrophages infected or not with *Helicobacter pylori* (Hp), upon activation of CD300E with the agonistic monoclonal antibody; as control, macrophages were exposed to isotypic IgG (control IgG). Data are shown as mean fluorescence intensity (MFI) ± SD of three independent experiments performed with three different cell preparations. **(C)** Impact of CD300E activation on the antigen presentation ability of macrophages to T lymphocytes. Data are expressed as percentage of proliferating cells as determined by CSFE-based staining (mean ± SD). Two independent experiments were performed with 10 different T cell clones for each experiment. Significance was determined by Student’s *t*-test. *p < 0.05; **p < 0.01; ***p < 0.001.

To determine the impact of CD300E activation in macrophages infected by Hp, we took advantage of an agonistic soluble anti-CD300E monoclonal antibody (UP-H2), being the natural ligand still unknown (20). Upon a 48 h infection, macrophages were incubated with the antibody and after a short (3 and 6 h) and a long incubation (24 h), mRNA expression profile was evaluated. The short time activation elicited a pro-inflammatory profile in macrophages, testified by the activation of pathways related to inflammation, as detailed in Table 1. In accordance, the activation of CD300E triggered the release of IL-1β and IL-6 by macrophages (Figure 7A). Interestingly, this activation, which was maintained at 24 h of infection (Table 1), was paralleled by the abatement of the MHC-II-dependent pathway of antigen presentation (Table 2). In particular, we found that mRNAs encoding for *HLA-DMA, HLA-DMB, HLA-DOA, HLA-DPA1, HLA-DPB, HLA-DRB1, HLA-DRB3, HLA-DRB4*, and *HLA-DRB5* were strongly downregulated (Figure S1 in Supplementary Material).

**Table 1:** Pathways activated upon CD300E activation.

Pathway name	FWER *p*-value
**3 h CD300E activation**	
PID_NFAT_TFPATHWAY	0.000
PID_TCR_CALCIUM_PATHWAY	0.003
PID_CD8_TCR_DOWNSTREAM_PATHWAY	0.011
PID_IL6_7_PATHWAY	0.012
BIOCARTA_INFLAM_PATHWAY	0.016
**6 h CD300E activation**	
BIOCARTA_INFLAM_PATHWAY	0.000
PID_NFAT_TFPATHWAY	0.002
**24 h CD300E activation**	
REACTOME_CHEMOKINE_RECEPTORS_BIND_CHEMOKINES	0.003
KEGG_CYTOKINE_CYTOKINE_RECEPTOR_INTERACTION	0.011
PID_TCR_CALCIUM_PATHWAY	0.025
PID_NFAT_TFPATHWAY	0.031

*On the left, the pathway name as indicated in the GSEA used databases, and on the right, the family-wise-error rate (FWER)*.

**Table 2:** Pathways inhibited upon CD300E activation.

Pathway name	FWER *p*-value
**24 h CD300E activation**	
KEGG_ANTIGEN_PROCESSING_AND_PRESENTATION	0.000
KEGG_LYSOSOME	0.028
REACTOME_MHC_CLASS_II_ANTIGEN_PRESENTATION	0.047

*On the left, the pathway name as indicated in the GSEA used databases, and on the right, the family-wise-error rate (FWER)*.

To validate the impact of CD300E on the modulation of the MHC-II pathway, we determined the expression of HLA-DR on the plasma membrane of macrophages exposed to the agonistic antibody. A 24-h stimulation of CD300E reduced the surface exposure of HLA-DR molecules by ~50% in uninfected macrophages (Figure 7B). Surprisingly, the infection of macrophages by Hp led to more than 50% drop of HLA-DR, and this occurred regardless of the antibody stimulation. However, a further decrease by 10% was detected upon anti-CD300E activation (Figure 7B). Such a decreased expression of HLA-DR on macrophages resulted in the impairment of the antigen presentation ability to T lymphocytes, as revealed by a proliferation assay (Figure 7C). Notably, the activation of CD300E affected the antigen presentation independently from the infection, in accordance to the HLA-DR expression pattern (Figures 7B,C).

Interestingly, despite the pathway of antigen presentation was compromised by the activation of CD300E, phagocytosis was stimulated by the activation of the receptor (Figure S2 in Supplementary Material), according to expression data (Figure S3 in Supplementary Material).

## Discussion

*Helicobacter pylori* infection continues to be a major public health issue worldwide and despite more than 30 years of research, after the discovery of the bacterium, there are still many unanswered questions. For example, although following successful colonization of the gastric mucosa by Hp, both branches of the host immune response are stimulated, namely, the innate and the adaptive one, it remains to understand how Hp escapes the immune surveillance, leading to a long-term infection and inflammation.

With this study, we demonstrated that Hp modulates the expression of several miRNAs in macrophages, some already identified as important for the response to Hp infection such as, miR-155, -223, -146a, and let-7 (21). Focusing on downregulated miRNAs, we revealed that they are permissive for inflammatory processes. Among genes upregulated involved in inflammatory processes we found that the main upregulated gene is that encoding for the immune receptor CD300E. CD300E belongs to a seven members’ family of immune receptors that includes activating and inhibitory members. Depending on the type of receptor, it can be expressed on myeloid cells, on lymphoid cells or on both compartments (22). CD300E is expressed on the plasma membrane of monocytes and its engagement by an agonistic antibody results in the expression of activation markers (i.e., CD83 and CD86) and secretion of pro-inflammatory cytokines (17). Notably, the differentiation of monocytes toward macrophages is accompanied by the downregulation of the expression of CD300E (20), the ligand of which is still unknown.

Infection of macrophages by Hp turns the expression of the receptor back on through the modulation of miR-4270. Macrophages expressing CD300E accumulates in the gastric mucosa of Hp-infected subjects with chronic gastritis while its expression drops down upon bacteria eradication. Notably, CD300E is not appreciable in the gastric mucosa of Hp-negative chronic gastritis, hence its upregulation can be considered a hallmark of Hp infection. In accordance, the exposure of macrophages to a non-pathogenic bacterium did not impact on CD300E expression.

The engagement of CD300E in infected macrophages leads to the release of pro-inflammatory cytokines and phagocytosis, but it reduces the exposure of MHC-II molecules on the plasma membrane. This effect is much more valuable in macrophages that have not been exposed to Hp due to the fact that the bacterium *per se* has a strong effect on the expression of the molecules of antigen presentation.

Based on these results, we propose the following scenario: during the first stage of infection, an adaptive immune response toward Hp is elicited; meanwhile, bacteria proliferate and impair the ability of macrophages to present the antigens. This would initially occur by a direct action of the bacteria on macrophages, involving a mechanism that deserves further investigation. But this effect would be reinforced by the downregulation of miR-4270 and the subsequent upregulation of the immune receptor CD300E. In fact, the engagement of CD300E by a ligand, which remains to be determined, would maintain the inflammatory status because of the release of cytokines, but it would also maintain the inability of macrophages to present bacterial antigens to T cells, thus impairing their local proliferation and the possibility for them to exert the effector function, such as the activation of the killing potential of macrophages through IFN-γ.

Collectively, our results reinforce the idea that Hp-infected macrophages may have a pivotal role in the persistence of Hp in the host, not only because the normal process of phagosome maturation is altered (23) but also because they become invisible to effector T cells, thus jeopardizing the possibility to clear the infection.

Moreover, our findings contribute to understand the function of the immune receptor CD300E. Considered until now a receptor expressed on monocytes and dendritic cells, we demonstrated that it can be expressed in macrophages upon the downmodulation of miR-4270. In addition, albeit ascribed to the activating members of the CD300 family till now, it actually elicits a more articulated immune response. Considering the importance of antigen presentation, studying CD300E and identifying its ligand(s) could unravel new mechanisms of immune escape not only in chronic infectious disorders but also in diseases in which an unproductive adaptive immune response can profoundly impact their outcome.

## Experimental Procedures

### Monocyte Isolation, Macrophage Differentiation, and Cell Treatment

Monocytes derived from buffy coats obtained from healthy blood donors were prepared as described previously (24). For macrophage differentiation, 5 × 10^5^ monocytes, seeded in 24-well plates, were cultured in RPMI 20% FBS in the presence of 100 ng/ml M-CSF (Miltenyi Biotec) for a 6-day differentiation. Cells were infected with bacteria (5 × 10^6^ CFU/ml, MOI = 10) in RPMI 1640 20% FBS. For comparing the impact of Hp on CD300E protein expression with that of *E. coli*, cells were infected for 6 h, washed with RPMI 20% FBS, and incubated for further 18 h in culture medium plus gentamicin (50 µg/ml).

### Bacteria

*Helicobacter pylori* strain N6 (kindly provided by Prof. C. Josenhans, Hannover, Germany) was maintained in 5% CO_2_ at 37°C on Columbia agar plates supplemented with 5% horse blood (ThermoFisher). Colonies were taken directly from plates and resuspended in RPMI 20% FBS without any antibiotic. Bacterial count was performed by determining the optical density (OD) at 600 nm (1 OD = 10^9^ CFU/ml). Before proceeding with the infection experiments, bacteria motility was verified at the optical microscope. One milliliter of a liquid culture of *E. coli* (strain K12/DH10B) was harvested, and cells were suspended in RPMI 20% FBS without any antibiotic. Bacterial count was performed as above.

### Flow Cytometry

Cells were harvested from culture plates using 5 mM Na–EDTA in PBS pH 7.5 and incubated for 15 min at RT with 10% human serum to saturate Fc receptors. 5 × 10^5^ cells were stained with a monoclonal antibody anti-CD300E (clone UP-H2, Abcam), followed by a goat anti-mouse Alexa Fluor 488 antibody (ThermoFisher). The cell viability dye eFluor780 (ThermoFisher) was used to exclude dead cells from the analysis. Cells were resuspended in FACS buffer (PBS, 1% BSA) and analyzed by a six-color FACSCanto II (Becton Dickinson). Forward and side scatter light were used to identify cell populations. Values were expressed as the ratio of the mean fluorescence intensity (MFI) of CD300E over the MFI of the secondary antibody. All data were analyzed using FlowJo software, version 10.3 (Tree Star Inc.).

### Ethics Statement

Investigation has been conducted in accordance with the ethical standards, the Declaration of Helsinki, and national and international guidelines and has also been approved by the authors’ institutional review board (35842/AO/17).

### Patients

The cases considered in this study were retrospectively collected from the Surgical Pathology and Cytopathology Unit at the University of Padua (January 2003 to December 2011). All patients were white and native of the Veneto region, and they underwent endoscopy at Padua University Hospital.

The study was conducted on a total of 40 endoscopic biopsy samples obtained from different biopsy sets and all collected from oxyntic proximal mucosa (i.e., gastric corpus). The series included the following: (1) normal gastric oxyntic mucosa obtained from dyspeptic patients, as control (*n* = 10); (2) mucosa from Hp^−^ (negative) non-atrophic chronic gastritis (*n* = 10); (3) mucosa from Hp^+^ (positive) non-atrophic chronic gastritis, before Hp eradication therapy (*n* = 10); and (4) mucosa from the same patients as in (3), who underwent clinical and histological remission, after Hp eradication. Hp was assessed by histology (modified Giemsa staining) and confirmed by clinical history, rapid urease testing, and/or ELISA (Hp-specific IgG Abs; GastroPanel, Biohit HealthCare) (25). Two trained gastrointestinal pathologists, blinded to any of the patients’ endoscopic or clinical information, jointly assessed the original slides (H&E, Alcian blue–periodic acid Schiff, and Giemsa for Hp).

### Immunohistochemistry

Formalin-fixed paraffin embedded tissue sections were stained with a rabbit anti-CD300E polyclonal antibody (Sigma-Aldrich). On appropriate antigen retrieval (water bath at 98°C for 40 min in ethylenediaminetetraacetic buffer pH 8.0), reactivity was revealed using NovoLink Polymer horseradish peroxidase-linked (Leica Biosystems) followed by diaminobenzidine. Characterization of CD300E positive cells was performed by double immunohistochemistry. After completing the first immune reaction, the second was realized using a monoclonal primary antibody to CD163 (clone 10D6, ThermoFisher), visualized using Mach 4-AP (Biocare Medical), followed by Ferangi Blue (Biocare Medical) as chromogen. Quantification of CD300E-expressing cells was performed on at least five high-power fields on sections double stained for CD300E and CD163. Immunostained sections were photographed using the DP-70 Olympus digital camera mounted on the Olympus BX60 microscope, and the digital pictures (each corresponding to 0.036 mm^2^) were used for cell count. Values were expressed as the mean ± SEM.

### Western Blot

After 24 and 48 h from Hp infection, macrophages were lysed in Triton X-100 lysis buffer (20), and proteins were quantified by BCA protein assay kit (ThermoFisher), according to the manufacturer’s instructions. Equal amounts of proteins were resuspended in NuPAGE LDS sample buffer (Novex, Life Technologies) supplemented with 50 mM of DTT and denaturated for 5 min at 100°C. Samples were separated electrophoretically in NuPAGE Bis-Tris 4–12% polyacrylamide gel (Novex, Life Technologies), and proteins were subsequently transferred onto PVDF membranes (Amersham). Membranes were blocked with 5% non-fat milk in Tris-buffered saline (TBS, 50 mM Tris–HCl pH 7.6, 150 mM NaCl) containing 0.1% Tween20^®^ (Sigma-Aldrich), and antigens were revealed using a rabbit anti-CD300E polyclonal antibody (1:200, Sigma-Aldrich) and a monoclonal anti-HSP90 antibody (1:10,000, Origene). Blots were washed three times with TBS plus 0.1% Tween20^®^ and incubated for 1 h at RT with horseradish peroxidase-conjugated anti-rabbit (Millipore) or anti-mouse IgG secondary antibody (Novex, Life Technologies), respectively. Blots were developed with enhanced chemiluminescence substrate (EuroClone), and the protein bands were detected using ImageQuant™ LAS 4000 (GE Healthcare Life Science).

### Functional Assays

Macrophages were either infected or not with Hp for 48 h; cells were detached with 5 mM Na–EDTA in PBS pH 7.5, harvested and plated at a density of 2.5 × 10^5^ per well in 24-well plates previously coated with 10 µg/ml of the monoclonal antibody anti-CD300E (clone UP-H2, Abcam), for 3, 6, and 24 h. Samples were collected and stored for RNA extraction and analysis.

For the expression of HLA-DR, macrophages were either infected or not with Hp for 48 h; cells were harvested and plated at a density of 2.5 × 10^5^ per well in 24-well plates previously coated with 10 µg/ml of the monoclonal antibody anti-CD300E (clone UP-H2, Abcam) or control IgG (MOPC-21, Abcam). After 24 h, cells were collected and stained with a monoclonal antibody anti-HLA-DR APC (clone L243, BD Bioscience); the cell viability dye eFluor780 was used to exclude dead cells from the analysis. Cells were resuspended in FACS buffer (PBS, 1% BSA) and analyzed by flow cytometry. All data were analyzed using FlowJo software.

Phagocytosis assay was performed on macrophages, either infected or not with Hp for 48 h, after a 3-h stimulation with the monoclonal antibody anti-CD300E or with control IgG (as detailed earlier).

Cells were collected, counted, and diluted at 2 × 10^6^ cells/ml in RPMI 0.2% BSA; after 30 min at 37°C, cells were seeded on prechilled 96-well plate (10^5^ cells/well in 50 µl) and kept at 4°C for 20 min. 50 µl of pHrodo™ Green *S. aureus* BioParticles^®^ Conjugates (ThermoFisher) in RPMI 0.2% BSA (1 mg/ml) was added to the cells. Immediately after the addition of particles (time 0), cells from three wells were harvested, collected in FACS tubes, and washed once in cold PBS. Plate was transferred at 37°C and, after 15 and 30 min, cells from three wells per time point were harvested and processed as above. Phagocytosis was determined by flow cytometry. A gate was selected to identify green fluorescence positive cells (cells that engulfed the particles); MFI was obtained from the selected cell population, and data were analyzed by FlowJo software. Phagocytic Index was determined by multiplying the % of fluorescent cells with the MFI value for each sample. Data obtained at 15 and 30 min were normalized on those at time 0 and expressed as arbitrary units. Culture supernatants of infected or not infected macrophages, incubated for 24 h with the monoclonal antibody anti-CD300E or with control IgG, were collected for quantification of cytokine content by ELISA assay: specific kits for IL-6 (Immunotools) and IL-1β (eBioscience) were used following the manufacturer’s instruction.

### Generation of Tetanus Toxoid-Specific Cell Clones

Tetanus toxoid (TT)-specific T cell clones were obtained from peripheral blood of three healthy donors, as described previously (26). Twenty TT-specific T cell clones were selected for this study.

### CSFE-Based Proliferation Assay

5 × 10^4^ infected or non-infected macrophages were seeded in 96-well plates pre-coated with 10 µg/ml of the monoclonal antibody anti-CD300E or control IgG for 24 h. After this time, CFSE-stained T cell clones, in medium plus 0.5 µg/ml TT, were added into each well.

Briefly, each clone was harvested and washed two times with RPMI 10% FBS and once with PBS. 1 µl CFSE 5 μM/10^6^ cell/ml PBS (Celltrace CFSE cell proliferation kit, ThermoFisher Scientific) was added to each cell suspension; after a 20 min staining in the dark, at RT, cells were washed with RPMI 5% human serum, to inactivate CFSE, counted and seeded at the density of 10^5^ cells/well. The percentage of proliferating T cell clones in response to TT was determined by measuring the CFSE fluorescence by flow cytometry after 18 h of macrophages–lymphocytes coculture; 5,000 events were acquired for each sample.

### RNA Extraction

Total RNA from macrophages was extracted with TRIzol reagent (ThermoFisher) according to the manufacturer’s protocol. RNA was quantified using the NanoDrop 1000 spectrophotometer (Nanodrop). Total RNA integrity and content of miRNAs in each sample were assessed by capillary electrophoresis using the Agilent Bioanalyzer 2100 with the RNA 6000 Nano and the Small RNA Nano LabChips, respectively (Agilent Technologies). Only samples of total RNA with a RNA integrity number >7 and with a concentration of small RNAs <30% were used for miRNA or mRNA gene expression analysis.

### Microarray Expression Profiles

#### miRNA Microarray

miRNA microarray experiments were performed using the Agilent Human miRNA Microarray 8 × 60K platform (Agilent Technologies) based on miRbase V.19 where 2,006 human miRNAs are represented. 200 ng of total RNA was labeled using miRNA Complete Labeling and Hyb Kit (Agilent Technologies), according to the manufacturer’s protocol. Labeled RNA was hybridized onto microarray slides using a rotational oven at 55°C for 22 h.

#### mRNA Microarray

200 ng of total RNA was used for Cy3 labeling according to Low Input Quick Amp Labeling Kit (Agilent Technologies). SurePrint G3 Human Gene exp v3 microarrays (Agilent Technologies) were used to identify the genes modulated upon the activation of CD300E. Labeled RNA was hybridized onto microarray slides using a rotational oven at 65°C for 17 h.

After hybridization, both miRNA and mRNA microarray slides were washed and scanned in an Agilent microarray scanner (model G2565CA). Agilent Feature Extraction software version 10.5.1.1 was used for image analysis. Microarray data expression is available in the U.S. National Center for Biotechnology Information Gene Expression Omnibus (GEO) database under the following accession numbers: GSE98641 for miRNA analysis and GSE98639 for mRNA analysis upon CD300E activation.

### Microarray Data Analysis

#### miRNA Microarray Analysis

Raw microarray data were first filtered for the number of miRNAs presenting an expression value above background (allowed 50% of undetected values for each miRNA) and then normalized according loess cyclic algorithm (27). Sample clustering was performed according to miRNA expression of detected miRNAs. Pearson correlation and complete linkage were used for cluster analysis, while differentially expressed miRNAs were identified according to significance analysis of microarray (SAM) (28), maintaining a median false discovery rate (FDR) as 0%. Both analyses were performed using tMev software (29). miRNA function was inferred according to TAM tool (30), whereas miRNA targets were recovered by miRDB database (31). To identify functional miRNA targets, predicted miRNA targets were filtered out according to their inverse expression respect to miRNAs. Corresponding network was analyzed according to NetworkAnalyzer Cytoscape plug-in (32). BioGrid database v. 3.4 was used to identify functional relationship between different mRNAs described in the network. Node degree parameter was used to size nodes in the network.

#### mRNA Microarray Analysis

A meta-analysis approach was applied to identify functional miRNA targets based on the mouse microarray experiments from Weiss et al. (16). Raw microarray gene expression data were recovered from GEO database under the accession number GSE42622. Data obtained from infected and non-infected macrophages were quantile normalized, and probe expression values that did not pass filter (positive and significant) were set as NA (not available). Probes with more than 50% of NA were excluded from the analysis; this resulted in 29,338 analyzable probes. The same procedure was followed for the analysis of human microarray expression carried out to identify the genes that were modulated upon CD300E activation. 24,836 probes were used to evaluate the effects of CD300E activation. SAM test (28) was used to identify genes differentially expressed in mouse microarray. Mouse differentially expressed genes were converted to orthologous human genes using Biomart (33) and NCBI Homologene. Enrichment analysis of differentially expressed genes was performed according to DAVID web tool (34). Gene Set Enrichment Analysis (35, 36) was used to identify gene sets modulated by CD300E activation. To identify significant pathways, it was considered a family-wise error rate (FWER) lower than 0.05. FWER control exerts a more stringent control over false discovery compared with FDR procedures (35).

### qRT-PCR

TaqMan method was used to evaluate miRNA expression by qRT-PCR. 10 ng of total RNA was retrotranscribed using the TaqMan MicroRNA Reverse Transcription Kit (ThermoFisher), according to the user manual. qRT-PCR was performed using the 7500 Real-Time PCR System (ThermoFisher) in 20 μl using the Taq Man Universal PCR Master Mix II (ThermoFisher) according to the manufacturer’s protocol. PCR reaction was performed as follows: 50°C for 2 min; 95°C for 10 min; 95°C for 15 s, 60°C for 1 min, for 40 cycles.

For evaluating mRNA expression 1 µg of total RNA was retrotranscribed using the Superscript II enzyme (ThermoFisher) and oligod(T) as starting primer. Retrotranscription was performed at 42°C for 2 h in a thermostatic bath. After precipitation, 10 ng of cDNA was used in the qRT-PCR reaction performed in a 7900HT Fast Real-Time PCR System (ThermoFisher). qRT-PCR reaction was performed in 10 µl using SYBR Green master mix (ThermoFisher), according to the following cycle: 95°C for 5 min; 95°C for 15 s, 60°C for 1 min, for 40 cycles. For each sample, data were normalized to the endogenous reference gene (*GAPDH*). Uninfected cells were taken as the reference value, and the relative expression was calculated. Data analysis was carried on according to ΔΔCt method for both miRNA and mRNA. Primer sequences were included in Table S8 in Supplementary Material.

### Luciferase Assay

Macrophages were transfected with miRNA mimics and 100 pg/µl of pmirGLO Dual-Luciferase miRNA Target Expression Vector (Promega) containing target or control sequences (Table S9 in Supplementary Material). Assays were performed using the Dual-Luciferase Reporter Assay (Promega) and measuring firefly and renilla luciferase activities in a Turner Designs TD-20/20 Luminometer (DLReady™, Promega). miRNA transfections were replicated independently at least three times.

### Statistic

Data are reported as the mean ± SD or mean ± SEM. The Student’s *t*-test and Mann–Whitney *U* test were used for statistical analysis of the differences between experimental groups. *p*-Values ≤0.05 were considered significant.

## Author Contributions

MP differentiated macrophages, infected them with Hp, and performed cytometric and miRNA qRT-PCR analyses; FM performed mRNA qRT-PCR analyses and phagocytosis assays; MT performed western blot and cytometric analyses; SL performed immunohistochemistry experiments; FC performed luciferase assays; GC performed phagocytosis assays; CM and BP performed microarray experiments; CDB performed assays for antigen presentation ability of macrophages to T lymphocytes; WV analyzed immunohistochemistry; MF collected and analyzed patients; MdB conceived the study and wrote the manuscript; SC analyzed microarray data, conceived the study, and wrote the manuscript.

## Conflict of Interest Statement

All the authors declare that the research was conducted in the absence of any commercial or financial relationships that could construe potential conflict of interest. The reviewer LE and handling editor declared their shared affiliation.
