# Supplementary material for: Helicobacter pylori Affects the Antigen Presentation Activity of Macrophages Modulating the Expression of the Immune Receptor CD300E through miR-4270
Source: Front Immunol. 2017 Oct 12;8:1288. doi: 10.3389/fimmu.2017.01288 (PMC5649134; doi:10.3389/fimmu.2017.01288)

Supplementary Material

# *Helicobacter* *pylori* affects the antigen presentation activity of macrophages modulating the expression of the immune receptor CD300E through miR-4270

Matteo Pagliari *^†^*, Fabio Munari *^†^*, Marta Toffoletto *^†^*, Silvia Lonardi, Francesco Chemello, Gaia Codolo, Caterina Millino, Chiara Della Bella, Beniamina Pacchioni, William Vermi, Matteo Fassan, Marina de Bernard ^#^ and Stefano Cagnin ^#^

^#^ Co-corresponding Authors

*^†^* Co-first authors

Correspondence: Marina de Bernard, Department of Biology, University of Padua, Padua, Italy; Tel: +39.049.827.6309; email: marina.debernard@unipd.it and Stefano Cagnin, Department of Biology, University of Padua, Padua, Italy; Tel: +39.049.827.6162; email: stefano.cagnin@unipd.it

# Supplementary Figures

**Supplementary Figure 1.** **Expression of genes of the MHC-II locus in Hp-infected macrophages upon CD300E activation.** Macrophages were infected for 48 h before activating CD300E by cell exposure to the anti-CD300E agonistic monoclonal antibody (clone UP-H2, Abcam) for 3, 6 and 24 h (3 h , 6 h, or 24 h anti-CD300E). Control cells (Ctrl), infected but not activated by the antibody, were harvested at the same time points. Gene expression is expressed referring to the average expression of the gene in all experiments. Histograms for each gene at each time point represent the average of gene expression of at least three biological replicates. Comparing with the control, genes for MHC-II were down-regulated at 6 and 24 h of CD300E activation.

**
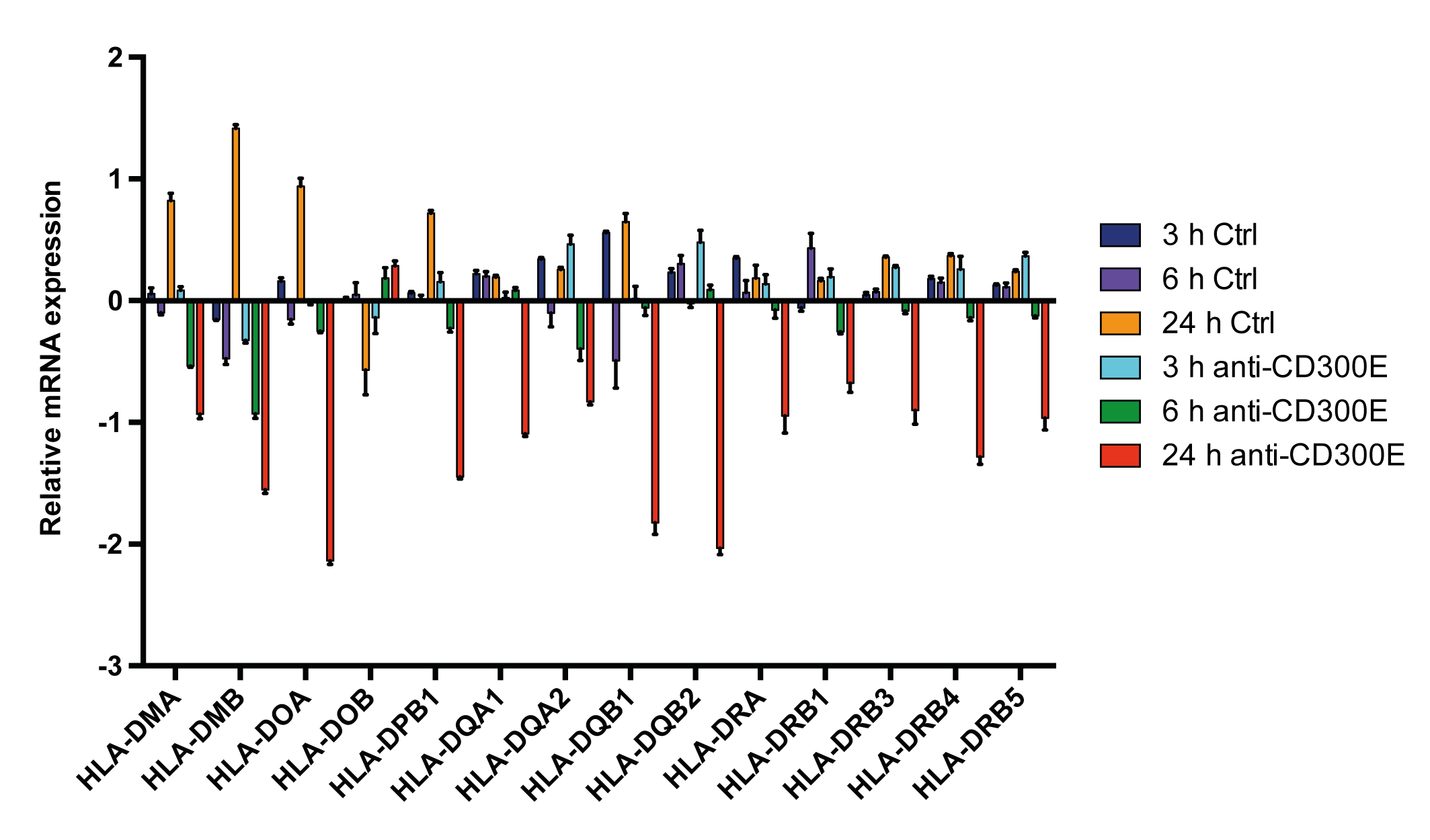
**

**Supplementary Figure S2. Impact of CD300E activation on the phagocytic process in macrophages.** Three independent experiments were performed with 3 different cell preparations. Significance was determined by Student’s *t*-test. ** p < 0.01.

**
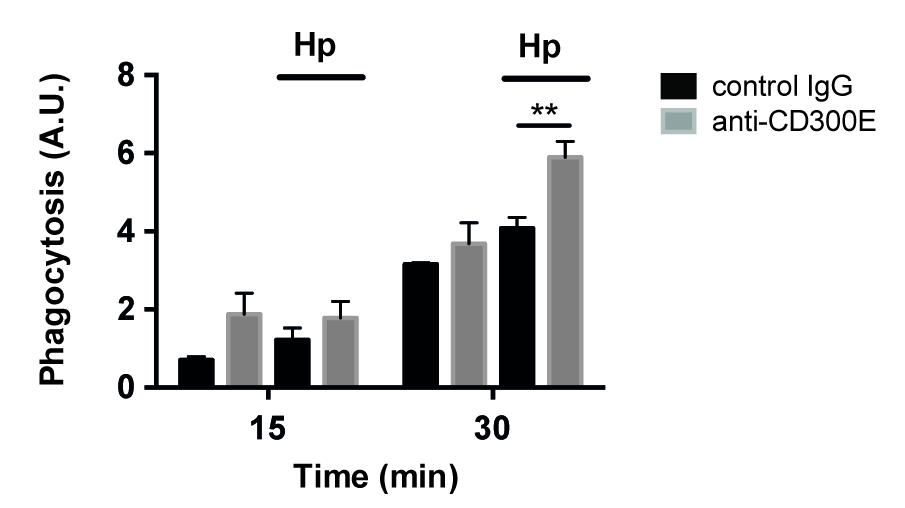
**

**Supplementary Figure 3. Heat map representing the expression of genes involved in the phagocytosis in infected macrophages upon CD300E activation.**  Macrophages were infected for 48 h before activating CD300E by cell exposure to the anti-CD300E agonistic monoclonal antibody (clone UP-H2, Abcam) for 3, 6 and 24 h (3 h , 6 h, or 24 h anti-CD300E). Control cells (Ctrl), infected but not activated by the antibody, were harvested at the same time points. The expression of each gene is expressed referring to the average expression of the gene considering all samples. Gene categorization (on the right) was retrieved basing the Qiagen Pathway categorization (http://www.sabiosciences.com/rt_pcr_product/HTML/PAHS-173Z.html). Most of the genes are up-regulated after 3 h of CD300E activation.


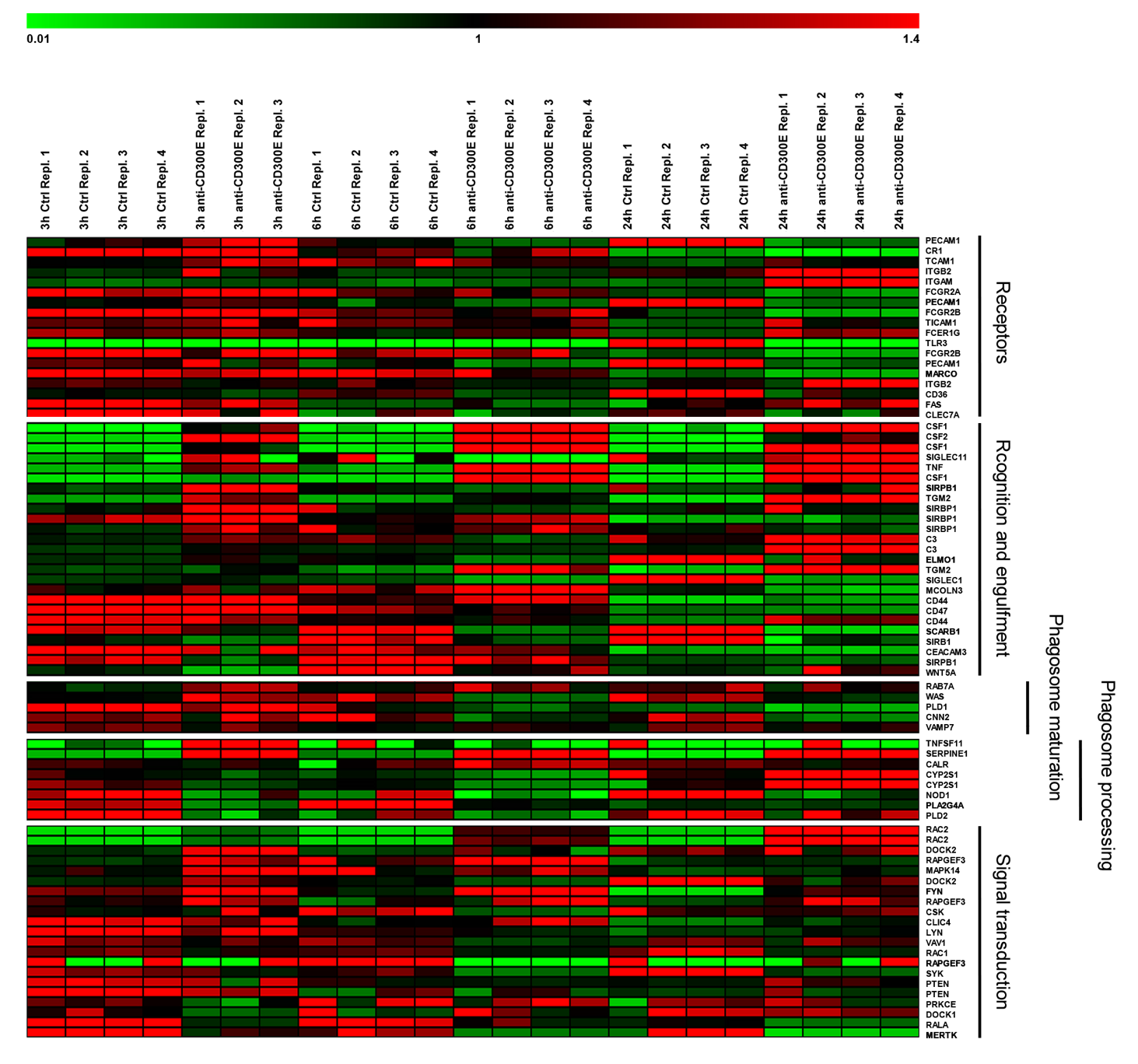

Supplement: Supplementary file 10 [file Data_Sheet_1.DOCX]
